# SMPPALD—Segmentation Mask Post-Processing Algorithm for Improved Lane Detection

**DOI:** 10.3390/s25196057

**Published:** 2025-10-02

**Authors:** Denis Vajak, Mario Vranješ, Ratko Grbić, Denis Vranješ

**Affiliations:** Faculty of Electrical Engineering, Computer Science and Information Technology, J.J. Strossmayer University of Osijek, 31000 Osijek, Croatia; denis.vajak@ferit.hr (D.V.); ratko.grbic@ferit.hr (R.G.); denis.vranjes@ferit.hr (D.V.)

**Keywords:** ADAS, lane detection, machine learning, computer vision, post-processing

## Abstract

As modern Advanced Driver Assistance Systems become increasingly prevalent in the automotive industry, Lane Detection (LD) solutions play a key role in enabling vehicles to drive autonomously or provide assistance to the driver. Many modern LD algorithms are based on neural networks, which estimate the locations of lane markings as segmentation masks in the input image. In this paper, we propose a novel algorithm, named SMPPALD (Segmentation Mask Post-Processing Algorithm for improved Lane Detection), designed to perform a set of post-processing operations on these segmentation masks to produce a list of points that define the lane markings. These operations follow geometric and contextual rules, taking into account the LD problem and improving detection accuracy. The algorithm was tested using the well-known and widely used Spatial Convolutional Neural Network (SCNN) on three different datasets (CULane, TuSimple, and LLAMAS). SMPPALD achieved a significant improvement in terms of F1 measure compared to SCNN on the TuSimple and LLAMAS datasets, while for the CULane dataset, it outperformed SCNN in most categories.

## 1. Introduction

Modern vehicles have a large number of systems that help the driver to drive efficiently and safely. These systems are named Advanced Driver Assistance Systems (ADAS), and they require various algorithms to function, as described in [1]. Many different problems in ADAS still need to be solved (e.g., [2,3]). One of them is the Lane Detection (LD) algorithm. LD is important because it allows multiple ADAS functions to work. These include lane departure warning and automatic lane following.

Most of the LD algorithms are based on processing the image captured by a front-mounted RGB camera on the vehicle, usually mounted where the center mirror is. LD algorithms can be split up into two different approaches, deep learning-based and computer vision-based, as proposed by [4]. Computer vision-based ones focus on image processing and analysis to obtain the necessary locations of lane markings on the road. Some examples include [4,5,6]. These solutions have been less common recently, in favor of deep learning-based approaches. Deep learning-based ones usually rely on a segmentation neural network (NN). The NN estimates the probability of each pixel belonging to a lane marking. The result is stored as a segmentation mask, i.e., a per-pixel probability map, which can be visualized as a grayscale image. This output is then processed by various algorithms to obtain the locations of lane markings. Some examples of these solutions include [7,8,9]. Although the deep learning-based solutions have achieved significant improvements in the problem of LD, outputs are often inconsistent and lack continuity in the case of occlusion, bad weather conditions, etc.

In this paper, a post-processing algorithm is proposed, dubbed SMPPALD—Segmentation Mask Post-Processing Algorithm for improved Lane Detection. SMPPALD is designed to improve the result of the segmentation NNs that predict the positions of lane markings in the input image. SMPPALD expects an input of four segmentation masks generated by the segmentation NN. It then applies a set of graphical and mathematical operations on them, which follow certain geometric and contextual rules. These rules include parallelism of the lane markings, standard minimum distance between the lane markings, continuity of the lane markings in the segmentation masks, maximum curvature of the lane markings, and maximum number of relevant lane markings on the road. The final output of SMPPALD is a list of points that belong to each of the four lane markings on the road. SMPPALD is designed to be used with any NN that outputs up to four segmentation masks and can be used to boost the results of any such solution.

Comparison of SMPPALD’s performance with the baseline model has shown significant improvement on three more popular datasets: CULane [7], TuSimple [10], and Llamas [11].

In Section 2, an overview of the current LD state of the art is given. In Section 3, SMPPALD is explained in detail. In Section 4, the process of training the segmentation NN and the estimation of the parameters of SMPPALD is described, as well as which datasets were used during performance evaluation. Results are given and compared with the results of another widely used solution. Finally, Section 5 concludes the results and findings.

## 2. Related Work

With the advancement of technology, modern transportation systems are becoming increasingly smarter. The increasing use of artificial intelligence, and, in particular, the use of deep learning methodologies, in smart vehicles, smart planning, and vehicle security, plays a key role in this process [12]. LD is one of the basic tasks that needs to be performed in smart vehicles.

To obtain a better understanding of the current state of the art in LD, recent overview papers are considered, as well as papers covering different approaches. At the highest level, they can be divided into computer vision-based and deep learning-based approaches, as proposed in [4]. Computer vision-based ones are less common today, but some will be mentioned later in this section. Most common solutions today, however, are deep learning-based approaches. The dominant methods nowadays are deep learning-based, which can themselves be categorized according to the classification proposed by the authors of [1]. The authors provide a review of recent advancements in LD, focusing on both 2D techniques and 3D methods. The authors split the various 2D LD methods into two classifications based on the number of stages required to complete them: Segmentation-based methods and Object detection-based methods. Segmentation-based can be mask-based, grid-based, or key point-based. These methods are two-stage and perform instance discrimination and lane localization sequentially. Object detection-based methods, on the other hand, are single-stage and perform instance discrimination and lane localization in parallel. Three-dimensional LD methods are split into two categories as well: birds-eye-view-based methods and birds-eye-view-free methods. The algorithm proposed by this paper is a 2D type, however, so that will be the primary focus of the related work.

Since the SMPPALD proposed in this paper requires a segmentation NN to be part of its processing steps, it is necessary to mention a few compatible examples. First such NN, named Spatial Convolutional NN (SCNN) [7], is used as a baseline to test against in this paper, as well as using it as the processing step. The authors propose an NN that generalizes traditional deep layer-by-layer convolutions to slice-by-slice convolutions within feature maps. This enables message passing between pixels across rows and columns in a layer. This makes it work well with long, continuous shape structures and large objects. SCNN outputs four grayscale segmentation masks, which can be perfectly used to perform computer vision-based processing to obtain lane estimation. This is considered one of the first such papers and is considered a baseline for many other papers. In addition to the proposed solution, a dataset dubbed CULane [7] was also created to test the solution. The results show that SCNN can learn the spatial relationship for structure output and significantly improve performance over other existing solutions.

Another segmentation NN, LaneAF [8], performs instance segmentation via embedding/clustering instead of fixed classes. It produces a binary segmentation mask of all lane markings plus an additional affinity field output. The affinity vectors are then used to cluster pixels belonging to the same lane, followed by vectorizing each lane marking. This results in separate lane instance masks without prespecifying the number of lanes. LaneAF was tested on both CULane [7] and Llamas [11] datasets and demonstrated better performance than other approaches, because of the model’s ability to detect and cluster lanes effectively and robustly.

Another paper proposes a hybrid feature-fusion strategy, dubbed as LHFFNet [9]. The first part of the approach uses multi-head self-attention to construct a multi-space attention enhancement module for feature enhancement in multispacer. This enhances the correlation of multiscale lane marking features. The second part of the solution involves a spatial separable convolutional branch for the jumping layer structure connecting multiscale lane marking features. Finally, strip pooling is introduced to refine the representation of lane marking information and optimize model performance. LHFFNet was tested on TuSimple [10] and CULane [7] datasets and has shown better results than most other approaches.

In [13], a simple NN named Ripple Lane Line Detection Network (RiLLD-Net) is proposed first. This network exploits quick connections and gradient maps to effectively learn lane marking features. This network can handle the most common LD scenarios. To handle more challenging scenarios, however, the authors introduce a more complex NN, named Ripple-GAN. This NN is created by integrating RiLLD-Net, confrontation training of Wasserstein generative adversarial networks, and multi-target semantic segmentation. This NN can handle complex scenarios such as occluded or complex lane markings and has shown better performance than other solutions when tested on the TuSimple [10] dataset.

Another approach proposed in [14], dubbed the ECBAM_ASPP model, integrates the Efficient Convolutional Block Attention Module (ECBAM) with the Atrous Spatial Pyramid Pooling (ASPP) module. ECBAM builds upon traditional attention mechanisms and employs dynamic convolution kernels to eliminate dimensionality reduction, enhancing the efficiency of feature channel learning and local interactions while preserving computational efficiency. This helps direct the network to focus on salient features while suppressing irrelevant ones. The model also performs variable sampling of the input, which achieves multi-scale feature extraction, enabling it to capture richer lane-related feature information. The model was tested on TuSimple [10] and CULane [7] datasets, with results showing superior overall performance.

A solution in [15] tackles the need for a faster approach when dealing with on-board hardware limitations, as well as issues when dealing with more complex scenarios. The authors propose an enhancement to the row-anchor-based LD method. Authors leverage the transformer encoder–decoder structure, as the row classification enhances the model’s capability to extract global features and detect lane markings in intricate environments. Additionally, a Feature-aligned PyramidNetwork (FaPN) structure is used as an auxiliary branch, complemented by a novel structural loss with expectation loss, which helps further refine the accuracy. The model was tested on the TuSimple [10] dataset and has shown exceptionally high framerate in addition to improved accuracy when compared to the baseline method.

Results of the segmentation must be processed for the data to be extracted from them. One such approach is proposed in [16]. This solution is a lane instance extraction algorithm based on prior knowledge and distance penalty modules and is aimed at improving the random sample consensus algorithm. The solution is made from two modules: The prior knowledge module and the distance penalty module. The first one uses existing lane prior knowledge to set filtering conditions in the algorithm iteration process, enhancing the robustness of instance extraction, while the second applies an evaluation penalty based on the distance of lane feature points, solving the deviation at the near end of the lane model caused by a sudden change in the number of feature points due to perspective transformation and improving the accuracy of instance extraction. Authors have tested the approach on a large amount of testing data from various scenarios and have shown that it can be applied to multi-LD post-processing tasks that can contain any number of lanes. The results show a high accuracy rate.

However, more similar to the solution proposed in this paper, other solutions exist that perform classical image processing on the segmentation masks to obtain the lane centerline or connected-component analysis to isolate each lane marking. These techniques are less common in newer papers, but one paper that can be mentioned is [17]. Here, the authors treat each lane as a separate instance, which can be trained end-to-end. In addition to instancing the lanes, authors also propose a learned perspective transformation, which automatically adjusts to the image, eliminating issues related to a fixed bird’s-eye-view transformation. The authors verified their approach on TuSimple [10], showing competitive results with other solutions.

In addition to these more complex deep learning-based approaches, purely computer vision-based solutions also exist, but are less common today. One such solution is presented in [4]. In the paper, a classic computer vision-based algorithm is proposed, dubbed HistWind2, which completely avoids the usage of NNs. The solution uses a combination of image processing techniques like perspective transformation, brightness and contrast adjustment, followed by histogram computation and a sliding window algorithm. HistWind2 achieved higher performance when compared with the original solution we were comparing against, but the improvement was mostly in execution time, showing some weaknesses of purely computer vision-based approaches.

Another approach proposed in [5] demonstrated a powerful end-to-end LD method using computer vision techniques. The authors first presented a minimalistic solution based on edge detection and polynomial regression, which worked only for straight lane markings. After that, the authors proposed an improved LD technique based on perspective transformations and histogram analysis, allowing both straight and curved lane markings to be detected. Simulations in different scenarios were used to show the superiority of the proposed approach.

In [6], a solution was proposed that uses an improved Hough Transform Method. The model is based on both an improved Hough transform and straight-line model reconstruction. In addition to the lane markings, the approach is also able to detect the vehicle in front, using preprocessing, vehicle shadow merging based on the improved search algorithm, regions of interest demarcation, lane determination, and vehicle tracking. Authors have shown a decent frame speed with a very high LD rate when driving on structured roads.

Since this area has been extensively researched in the last ten years, in addition to the papers analyzed in this section, there is a wealth of related research, such as those proposed in [18,19,20,21].

## 3. The Proposed Segmentation Mask Post-Processing Algorithm for Improved Lane Detection

To create an algorithm that is designed to accurately perform post-processing on segmentation masks created by segmentation NNs, certain constraints and specifics must be considered. Firstly, the output from segmentation NNs maintains the original perspective of the images from the original input, while lane markings on the road are generally parallel to each other. This is unsuitable, and a set of operations should be performed on the masks to make the lane markings parallel with each other. This results in the creation of the first segment of the algorithm, which can be named the Image Preparation segment.

After the lane markings are parallel, some kind of marking detection must be performed on them. The easiest way to detect the start locations of the lane markings is by using a histogram, where peaks correspond to markings. Once the start locations of the markings are found, another algorithm must be used to detect the full length of the markings. Experimentation during the design has shown that the sliding window algorithm works quite well for this, but it must be performed from both directions to obtain good results. The combination of these two algorithms forms the Marking Detection segment.

In the final segment of the algorithm, the found points must be further processed. The operations are not perfect and can sometimes result in multiple overlapping detections. A set of mathematically constrained algorithms had to be introduced, which further analyze the location of points and clean them up. This removes excess lane markings and overlapping points. This is the Point Generation segment. The output of this segment is the final output and can be used to compare against other solutions.

**Figure 1:**
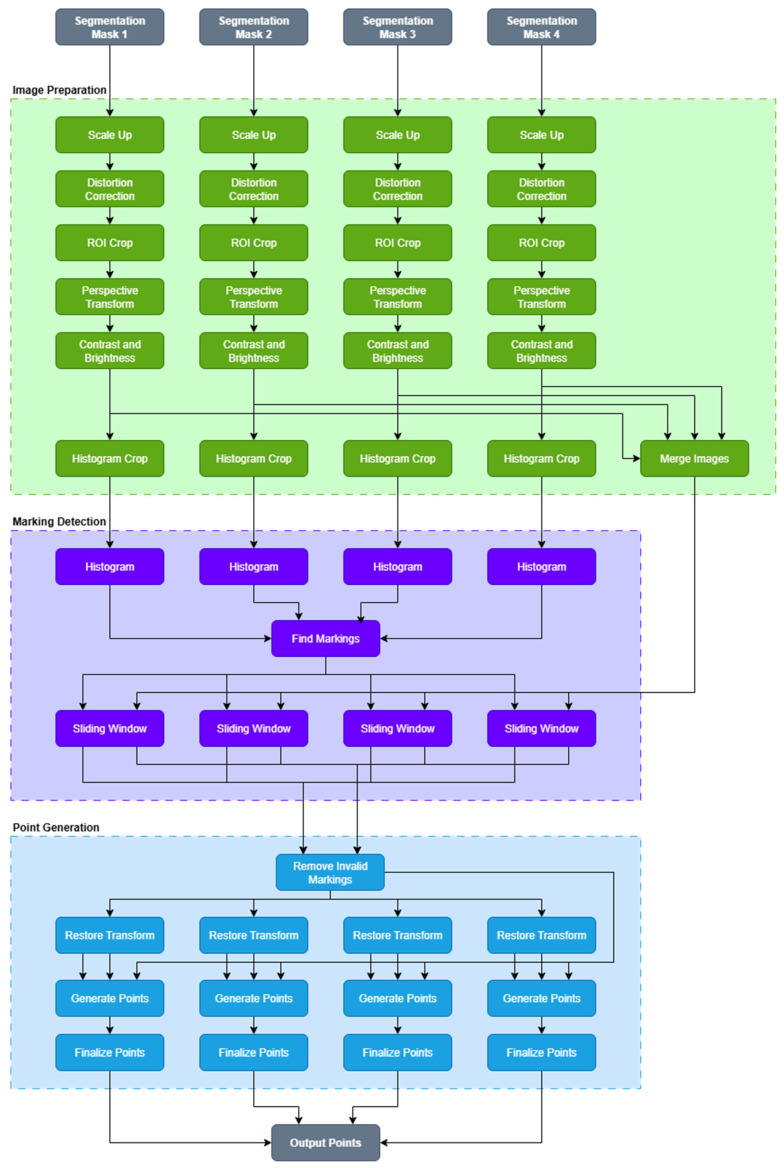
Functional block diagram of the proposed post-processing algorithm. The algorithm is split into three segments: Image Preparation, Marking Detection, and Point Generation.

Since the algorithm is designed to post-process segmentation masks and is used for LD, the algorithm was dubbed SMPPALD—Segmentation Mask Post-Processing Algorithm for improved Lane Detection. A functional block diagram of SMPPALD is shown in Figure 1.

Because all of the previously listed segments have various graphical, geometric, and mathematical parameters, Particle Swap Optimization (PSO) was introduced into SMPPALD. PSO helps estimate these various parameters, as doing this by hand is difficult and time-consuming. PSO allows the algorithm to effectively be “trained” for better results in every scenario using the same training datasets used to train the input NN.

### 3.1. Input Neural Network

**Figure 2:**
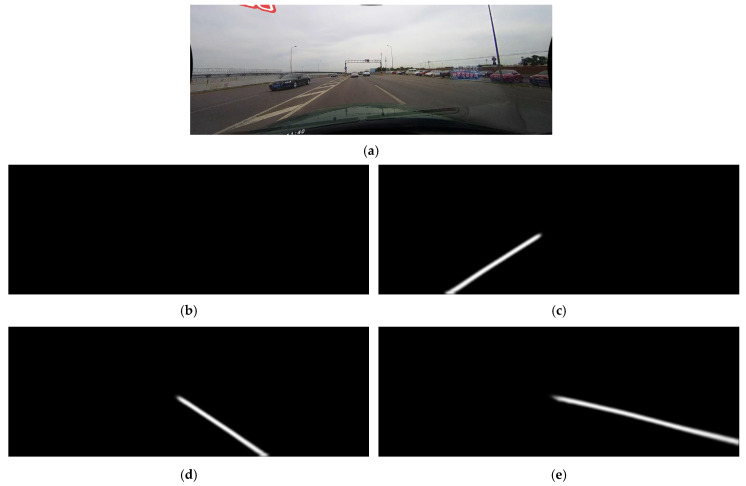
Original image from the CULane dataset [7], as well as the four segmentation masks generated by the NN. (**a**) Original image of the road from the CuLane dataset; (**b**) Segmentation mask corresponding to the far-left marking (missing because it is invalid in this situation); (**c**) Segmentation mask corresponding to the close-left marking; (**d**) Segmentation mask corresponding to the close-right marking; (**e**) Segmentation mask corresponding to the far-right marking.

The first step is based on using a segmentation NN of choice. The NN needs to generate four separate grayscale images, representing the four lane markings on the road. The grayscale level of each pixel represents the confidence level for that pixel to belong to a lane marking. For this paper, the well-known and widely used SCNN model proposed in [7] was used in this stage. An example input image, as well as the four generated segmentation masks, is shown in Figure 2. Figure 2a shows the original input image, while Figure 2b–e show the four segmentation mask outputs for the four lane markings from left to right. Segmentation mask output for the far-left marking (Figure 2b) appears empty, as there is no marking to be detected for it in this situation. The output of this model is then fed into the next stages of the algorithm.

### 3.2. Image Preparation Segment

This segment performs a set of simple image manipulations to prepare the four segmentation masks for use in the Marking Detection segment. The first step is scaling the images up. Because the NN runs on a resolution lower than the original images, segmentation masks must be scaled back up to their original size in the dataset. The output resolution of the NN depends on the configuration file of the NN and can be different for different datasets. Similarly, the target resolution is also dependent on the original resolution of the images in the used dataset, and this step will scale the images back up to the original resolution.

**Figure 3:**
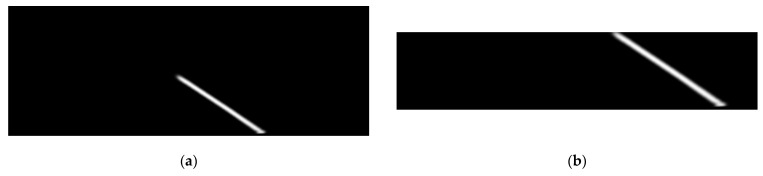

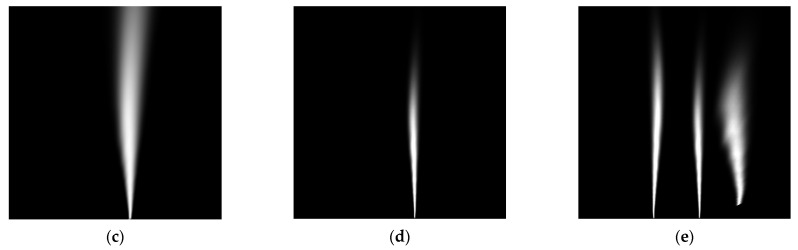
Results of processing steps during the Image Preparation segment of the algorithm, with steps performed on the close-right marking. (**a**) Lens corrected image for the input image presented in Figure 2d; (**b**) ROI cropped image after processing image presented in (**a**); (**c**) Perspective transformed image after processing image presented in (**b**); (**d**) Brightness, contrast, and gamma corrected image after processing image presented in (**c**); (**e**) Image created by merging the four outputs, one of which is presented in (**d**).

The next step is applying lens distortion correction. This is not always necessary, but some datasets like CULane [7] have a more noticeable lens distortion. This distortion can negatively affect the logic behind the Marking Detection segment of the proposed algorithm, so it is necessary to compensate for it. Lens distortion correction is performed using the standard pinhole camera model, separately for each dataset. The output of this step is visible in Figure 3a.

To keep only the useful area for the algorithm, a Region of Interest (ROI) crop is performed next. This ROI is dependent on the dataset, as it is influenced by things like camera position in the vehicle, angle of the camera, and field of view. Output is shown in Figure 3b.

Because the Marking Detection segment expects the lane markings on the road to be parallel with each other, and that is not feasible in a standard perspective of the road, perspective transformation must be performed. The perspective of the newly obtained ROI is transformed into a bird’s-eye-view perspective using bilinear interpolation. To obtain the perspective points of the source, a set of values is defined for each dataset. The first value is the X position of the center point closest to the camera. This value changes between datasets as it is dependent on how the camera was positioned within the vehicle while recording. An additional two values are needed, and these are the width of the road closest to the vehicle and the width of the road at the vanishing point. These values are also dependent on the dataset, as they depend on the camera’s field of view and the positioning. Lastly, a file is created in each image folder of the datasets, which defines the vanishing point for that set of images. In cases like with CULane [7], this value cannot be set globally for the whole dataset, as the camera could move between recording sessions. Vanishing point position is of the highest importance, as it ensures that the markings are parallel after the perspective transformation. No matter the source resolution, the output of the perspective transformation step is always 400 × 400 pixels, as the rest of the algorithm has some strict mathematical constraints that are built on this resolution. The result of the perspective transform is visible in Figure 3c.

It was shown that after the perspective transformation, the segmentation mask might become blurred. This could make it difficult for the Marking Detection segment to process, so the next step is to improve the sharpness. To do this, *contrast*, *brightness*, and *gamma* are adjusted. All three of these values are defined as parameters for the PSO. At this point, there are still a total of four images on which these operations were performed. Output is shown in Figure 3d.

These four images are then cropped further for the next segment of the algorithm. These new images are used for histogram calculations, and the height of the crop, measured from the bottom, is defined as the PSO parameter *histCrop*.

Additionally, a fifth image is created by merging the previous four outputs from the previous contrast, brightness, and gamma steps. To merge the images, the grayscale values of the pixels are simply added together, while ensuring that they do not go above the maximum value of 1. This merged image is used for the later sliding window steps. The merged image is visible in Figure 3e.

### 3.3. Marking Detection Segment

This segment uses mathematical algorithms to perform initial detection of the lane markings in the previously processed images.

**Figure 4:**
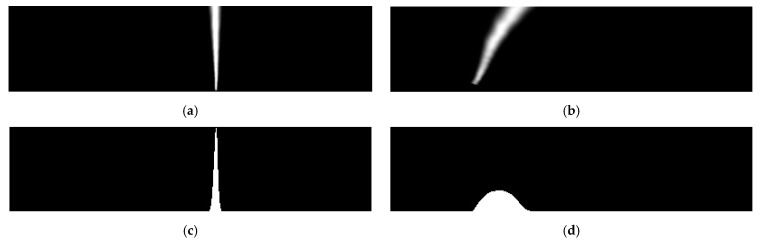
Graphical representation of the histogram computation: (**a**) Example input cropped image with a straight lane marking; (**b**) Example input image with a curved lane marking; (**c**) Calculated peak using histogram for straight lane marking; (**d**) Calculated peak using histogram for curved lane marking.

The first step is a histogram computation on all four of the cropped images. A sum value is calculated for each column. These sums create peaks, and each peak represents a possible marking. An example input and output of the histogram is shown in Figure 4.

**Figure 5:**
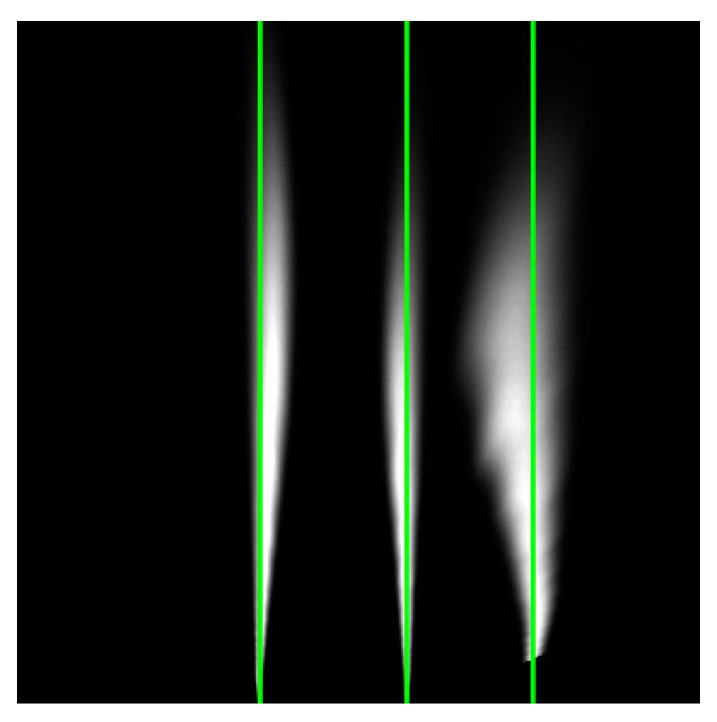
Visual representation of columns that contain lane marking start points (green lines), determined by the histogram output.

The output of these four histogram computations is then fed into an algorithm that analyzes them and compares them to determine which represent the lane markings and which should be rejected. The algorithm ensures that only four outputs are generated, corresponding to the original four lane markings. To achieve this, the algorithm looks for a certain histogram threshold above which a marking peak is detected, as well as a clash area. The clash area corresponds to several pixels to the left and right of the detected peak, where another peak should not be found. Both parameters are estimated using PSO and are defined as *histThresh* and *clashArea*, respectively. This helps prevent false detections. Figure 5 shows a representation of the output from this step.

The next step to be performed is the sliding window algorithm. This algorithm produces a set of points for each lane marking that correspond to that marking. This algorithm uses the previously merged image, as well as the found marking locations from the previous step. The algorithm is executed four times, once for each of the marking location outputs. Starting from the bottom of the image, at the location where the marking is expected, a window is designated for pixel analysis. The width and height of this window are determined by using PSO, as they can be significantly different in different datasets. They are defined as *windowWidth* and *windowHeight*, respectively.

Each window analyzes all the pixels within it to determine the X location of the marking in that window. This is performed by averaging all of the X locations where a pixel was found. Y location is always considered the middle of the height of the window. Because of the nature of the lane markings on the road, these windows need to move left and right along the X axis on each step of the algorithm, to follow the marking. A new window is created on the same X as the found marking in the previous window. Experimentation has shown that a maximum of 10 pixels works well as a constraint for how far the window should be allowed to move compared to the previous window.

Windows use a set of parameters to determine if they should be considered as valid or not. The first such parameter is *thresholdFirst*. This parameter defines the minimum needed threshold for a window to be considered at the bottom start of the marking. For regular windows, *thresholdMin* is used instead. Both of these parameters have the *thresholdDip* parameter subtracted from them. If the threshold is fulfilled, the window will analyze the pixels. A new threshold is automatically determined by taking the highest value of pixels in the window and subtracting the *thresholdDip.* All found pixels are added to a list, and if enough pixels are found, based on the *minLineDots* parameter, the middle X point of all pixels is computed. All these parameters are adjusted by PSO.

The sliding window algorithm also tries to determine outliers. These are windows that are skipped because the found markings in them are outside of the expected X distance, for instance, if there is a curved marking and the window hits another marking. The starting window has these limits set to a higher value using the *outlierFirst* parameter. If a window is designated as valid, the *outlierStart* parameter is used instead. This parameter is used until there is a skipped window, for instance, due to some artifact of the segmentation. If a window is skipped, the outlier distance is incremented by the *outlierIncr* parameter for each new skipped window until a new window is found. The reason for this is that a curved marking might be significantly further away than expected. These outlier parameters are also adjusted by the PSO.

**Figure 6:**
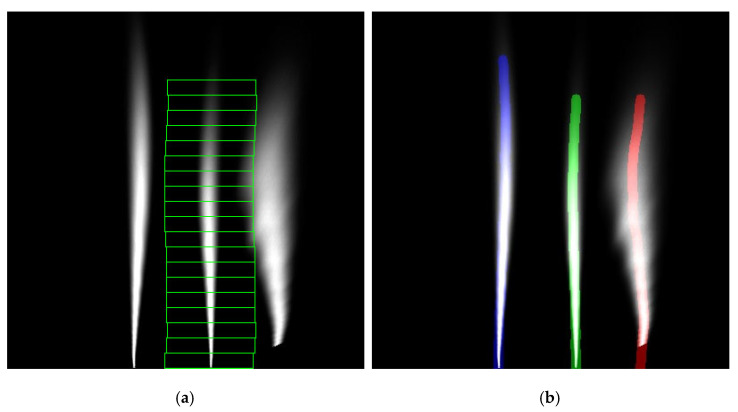
Results of the sliding window algorithm. (**a**) Windows determined along the length of the close-right marking, performed on the image presented in Figure 3e; (**b**) Visualization of curves made from points found by the sliding windows. Different detected lane markings are represented by different color lines.

Once the sliding window algorithm has reached the top of the image, it performs the same algorithm downwards. This is performed to fill in windows that it has possibly missed. Each found window corresponds to a single point of the marking. In case there are missing points at the bottom, which can happen in certain placements of the camera in the dataset, the algorithm will attempt to generate missing points by extending the marking at the bottom where it ends. It uses 2 points to generate a straight line to the bottom of the image, filling in missing windows. Figure 6a shows an example result of the sliding window algorithm.

As mentioned earlier, the sliding window algorithm is run on all four of the output marking locations. It outputs a list of points for each sliding window location as well as edge hit locations. These are locations where the sliding window has reached the edge of the image. With this, the points of the markings have been found. Figure 6b shows the final output of all four of the sliding window algorithms. The markings shown in the image are only for visualization purposes and were created by connecting the points into a curve. This applies to other images shown below as well. The next step is to further process the point lists to clean up the output.

### 3.4. Point Generation Segment

The final segment of the algorithm performs a set of cleaning operations on the points found by the sliding window algorithms, as well as preparing them for the final output.

**Figure 7:**
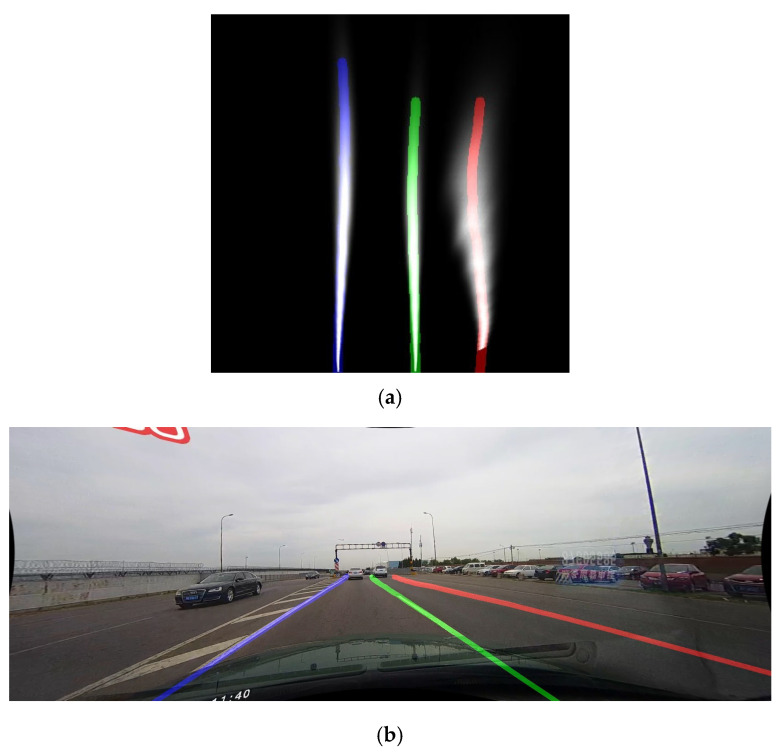

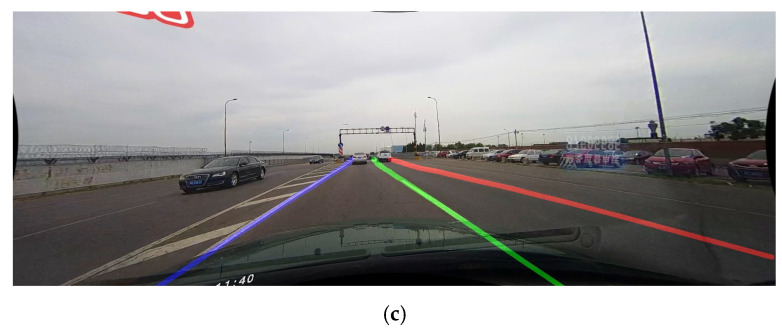
Outputs of the various steps of the Point Generation segment. (**a**) Visualization of the output after removal of invalid markings; (**b**) Visualization of the output after restoring the original perspective, with the original image added below the found markings; (**c**) Visualization of the output of the point generation step, with the original image added below the found markings. Different detected lane markings are represented by different color lines.

The first step uses an algorithm that removes invalid lane markings by comparing markings with each other. A point from both markings is chosen using the PSO parameter *overlapCheck*, and the distance between these two points along X is compared to the *xLineOverlap* PSO parameter. If they are too close together, one of the two markings is considered invalid. After that, a new set of markings is saved, while ensuring that there are no more than four lane markings. Both points and edge hits are kept for each marking. The output of this step is shown in Figure 7a, but in this example, there were no markings to reject, which is otherwise possible.

After this, the transformation of the points is restored to match the original image from the camera. Both the perspective transformation is computed in inverse, as well as camera distortion. This is performed separately for each marking. The output of this step is visible in Figure 7b.

Each marking’s points are then properly generated. Each point is first processed separately and eliminated if the point is within the PSO parameter *edgeDistance* from the left or right edge of the image. Because of the previous transformation, this step will also generate possible missing points in the marking, extending it all the way to the bottom of the image, as well as towards the upper part of the image. This is limited by the area of the initial ROI crop, but going slightly outside of the area, as defined with the PSO parameter *yLineExtraTop*. This upper extension will not be calculated if there are points above the PSO parameter *yLineOverTop*, or if this marking was marked as an edge hit previously. When performing these extension calculations, the PSO parameter *yLineSpacing* is used to decide what distance between points is used to pick two points when generating a straight-line extension. Figure 7c shows the output of this step. Figure 7b and Figure 7c might appear nearly the same, but the markings were slightly extended towards the vanishing point, and they had points that were outside of the image removed.

**Figure 8:**
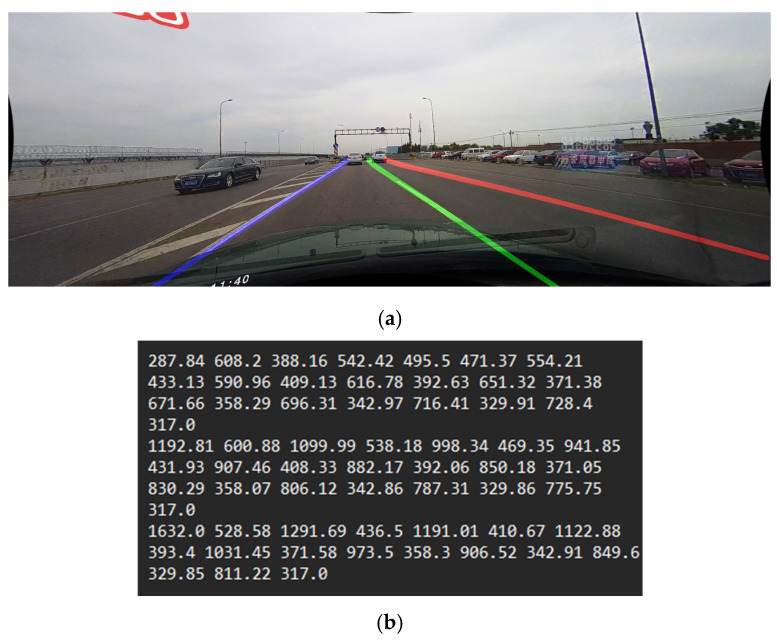
Final outputs of SMPPALD. (**a**) Visualizations of the final output, with the original image added below the found markings; Different detected lane markings are represented by different color lines. (**b**) Contents of the text output file.

Finally, all of the markings are processed by removing any large congestion of points. This happens because of the perspective transformation, causing the farther-away points to be significantly denser. Using the PSO parameter *yMinDistance*, points are analyzed, and those that are below that distance along Y are removed. This final output is visible in Figure 8a.

Final points are saved in a text file, where each lane marking is a new text line, and X and Y values for each point are listed one after each other with a space between them. This is a standard format accepted by the lane evaluation used by SCNN in [7], and an example output is visible in Figure 8b.

## 4. Results and Discussion

To determine the effectiveness of the algorithm in real scenarios and in various situations, the algorithm was tested on a total of three different commonly used datasets: CULane [7], TuSimple [10], and Llamas [11]. CUlane consists of various driving scenarios, including urban situations, highway situations, rural situations, as well as night and day scenarios, but has only 1 frame per second. It contains training, validation, and testing sets. TuSimple consists only of highway situations during the day and has a smaller total frame count, but unlike CULane, it has a higher framerate at 20 frames per second. It also contains training, validation, and testing sets. Llamas is similar to TuSimple; however, it has more frames and contains ground truth for only the training and validation sets, so the validation set was used for testing as well in this case. Table 1 shows an overview of resolutions and frame counts in the three datasets. CULane additionally has the testing set split up into nine categories, and these are shown in Table 2.

The first step consisted of training the SCNN using the dedicated training sets of each dataset. After each 10,000 batches, the trained model was used on the corresponding validation set to verify the success of the training. If either the Intersection Over Union (IOU) or accuracy was higher than in the previous validation step, a snapshot of the model was saved, as well as a label indicating whether the IOU or the accuracy was highest for that snapshot. Training was executed for a maximum length of 4,000,000 batches before it was automatically terminated, and the batches with the highest accuracy were compared against the testing sets with the CULane and TuSimple datasets, and the validation set for the Llamas dataset. To perform the comparison, the original MATLAB R2018b script provided by the SCNN authors, proposed in [7], was used, followed by the provided C script for lane evaluation. From the best IOU and best accuracy batch, the one with the best F1 measure was picked for each dataset. This provided a baseline both for the comparisons and as a starting point for training the parameters in the post-processing algorithm.

Non-constrained PSO was used on a total of 22 parameters, and a full list of them is in Table 3, along with descriptions and their numerical types. The estimation process needed to be performed only initially as part of the training, and the parameters were afterward loaded from a static parameter file. For estimation, the swarm size of the PSO was set to 50, and the total number of iterations was set to 25. At first, expected ranges for estimation were defined, and later experimentally adjusted after a few PSO iterations for each of the three datasets. Final ranges are listed in Table 4. In case some of the PSO parameters reached their limits, estimation was repeated with their ranges adjusted for a better expected fit. Final ranges of all the parameters, in relation to the datasets, are listed in Table 4. Parameter estimation was performed separately for each of the datasets, as the sets have varying camera parameters. Once estimation was complete, SMPPALD was executed on the testing sets with the CULane and TuSimple datasets, and the validation set for the Llamas dataset to obtain the final results.

Table 5 shows the results for all datasets. When results are compared with the post-processing approach of the original SCNN [7], SMPPALD achieved higher performance in most categories of the CULane dataset. Specifically, it has achieved higher performance in more difficult categories, including Crowd, No Line, Shadow, Arrow, and Dazzle. In the case of No Line, it has outperformed the original dataset by as much as a 0.031 F1 increase. The crossroad category is special in the way that the algorithm is supposed not to find any lane markings, as the crossroad is an open road. Thus, this category uses False Positives (FP) as a metric, where a lower number is better.

When tested on the TuSimple and Llamas datasets, the results are also higher than when using the post-processing from SCNN [7]. With the TuSimple dataset, F1 measure results are 0.028 higher, and with the Llamas, they are 0.023 higher, achieving better results in both.

When it comes to executional performance, it is important to mention the hardware used in this research. SMPPALD was tested on an AMD Ryzen 5 1600 CPU. Table 6 shows a comparison of frame processing execution time for each of the three datasets, providing the shortest, longest, mean, and median execution times. In the case of CULane and TuSimple datasets, testing sets were used, while the validation set was used for the Llamas dataset. Testing has shown that frame processing execution time can vary significantly based on the scene. Because of the way SMPPALD’s sliding window stage works, it can finish processing swiftly for less-demanding situations or take a longer time if the markings have poor visibility. This is because of the various built-in checks and iterators, which ensure detection in more difficult scenarios.

In terms of mean frame processing execution time, the results show that SMPPALD is able to process around 9, 5, and 4 frames per second for CULane, TuSimple, and Llamas datasets, respectively. This confirms its usability in real-time driving applications, where it can provide new information regarding lane markings from 4 to 9 times per second. Note that these results are achieved without multithreading and other optimization techniques that could improve the real-time performance of SMPPALD.

**Figure 9:**
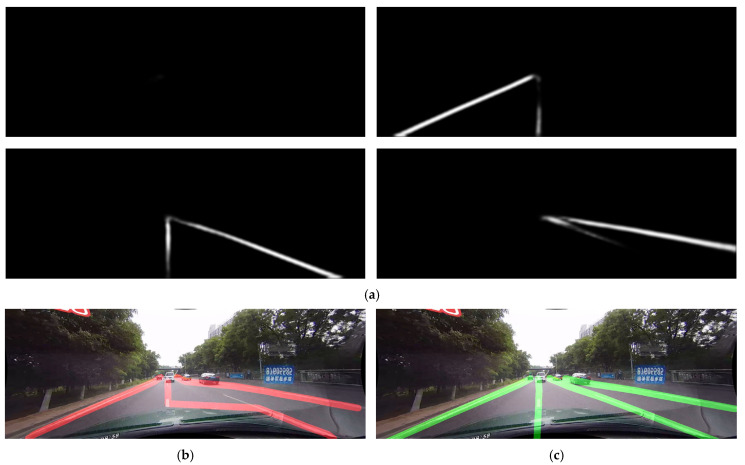
Example of comparison of SCNN post-processing vs. SMPPALD when switching lanes: (**a**) Segmentation masks showing ghosting of the lane markings; (**b**) SCNN post-processing output (red lines) showing misinterpreted lane markings; (**c**) SMPPALD’s output (green lines) showing a clean output and correct lane markings.

Deeper analysis of the results shows good examples of why SMPPALD performs better. SCNN was trained on mostly frames where the vehicle is driving in the middle of the lane, with lane markings to the left and right of the vehicle. When the vehicle switches lanes, segmentation masks begin giving incorrect output, as SCNN was not trained on that many lane switch frames. This results in ghosting of the lane markings in the output segmentation masks, which is visible in Figure 9a. Post-processing algorithms like SCNN’s approach have trouble processing these situations, which results in Figure 9b. Thanks to SMPPALD’s Image Processing steps and the complex sliding window algorithm, it can correctly identify these markings, as shown in Figure 9c. This is because SMPPALD has more context of the scene, taking into account all four of the segmentation masks when detecting the markings. This example is from the Normal category in the CULane dataset, showing that even though the overall F1 measure is lower in this category, there are frames where SMPPALD excels.

**Figure 10:**
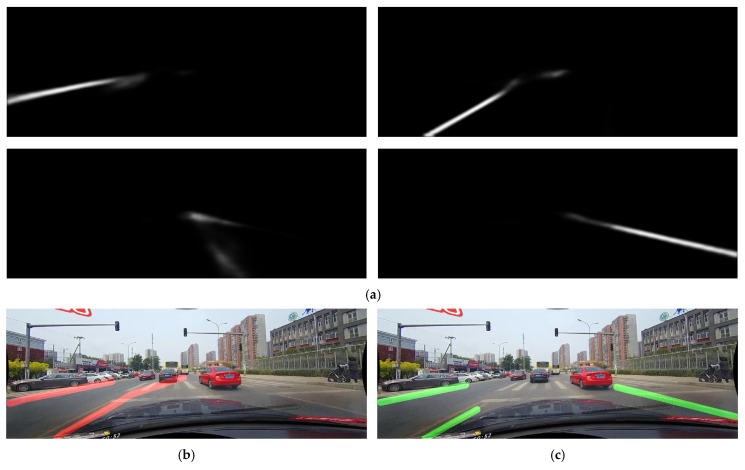
Example comparison of SCNN post-processing vs. SMPPALD on pedestrian crossings: (**a**) Segmentation masks; (**b**) SCNN post-processing output (red lines) misinterpreting lane markings; (**c**) SMPPALD’s output (green lines) showing a clean output and respecting the pedestrian crossing.

Another good example is when handling markings like pedestrian crossings. While the segmentation masks in these situations look mostly correct, as shown in Figure 10a, SCNN’s post-processing approach struggles with them, as shown in Figure 10b. SMPPALD has shown to be able to correctly identify the lane markings in situations like this, even being able to prematurely stop the length of the lane marking as it reaches the pedestrian crossing, as visible in Figure 10c. This is both because of the previously mentioned full context of all four segmentation masks that SMPPALD has, as well as because of the way its sliding window algorithm can prematurely stop the marking in case of outliers. It was, however, unable to identify the close-right marking, as the presence of that marking is already barely visible in the segmentation masks.

**Figure 11:**
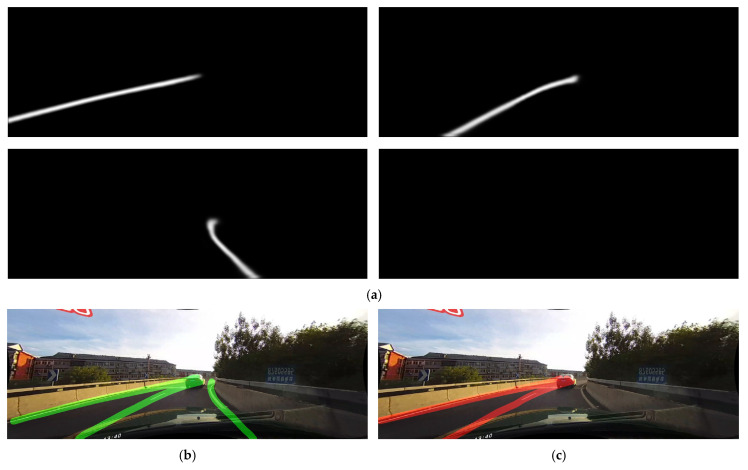
Example comparison of SCNN post-processing vs. SMPPALD on curved roads: (**a**) Segmentation masks; (**b**) SCNN approach’s output (green lines) detecting all three lane markings; (**c**) SMPPALD’s output (red lines) missing one lane marking.

However, SMPPALD has shown to have problems in certain curved roads. Because of the way PSO parameters are estimated on the entirety of the training set, most of the images include straight lane markings. This introduces a bias in the parameters, making them less suitable for curved roads. One way to solve this would be by using additional dataset, where curved roads would be a separate category during the training. In the example shown in Figure 11, SCNN’s post-processing approach was able to successfully detect all 3 of the visible lane markings, as shown in Figure 11b, while SMPPALD was able to find only 2, as shown in Figure 11c. This shows a clear point for improvement of SMPPALD in future work.

In summary, SMPPALD has shown the ability to improve the initial results of a chosen segmentation NN and increase the accuracy of lane marking detection. The proposed SMPPALD can be adapted in practice for each type of vehicle, taking into account the position and orientation of the camera in the vehicle. Thanks to the simplicity of the solution, it can easily be implemented on real ADAS as an upgrade to the existing segmentation NNs. Ultimately, his idea is not to replace lane marking detection solutions but to improve their performance, which increases the safety of all traffic participants.

## 5. Conclusions

In today’s modern vehicles, ADASs play an important role in helping the driver. Some ADASs rely on LD to determine where the lanes are located. There are multiple ways to categorize LD approaches as proposed in [1,4]. The proposed post-processing algorithm, SMMPALD, analyzes segmentation masks of an NN and performs a set of graphical and mathematical operations on them to determine the location of lane markings on the road. The algorithm aims to act as an easy-to-install upgrade to an existing segmentation NN, providing an easy improvement in its output. The algorithm uses PSO to estimate its parameters to achieve the highest performance. The SCNN was trained on 3 different datasets, and afterward, PSO parameters were estimated. A comparison was made with the original SCNN results against the results of SMMPALD, showing improvement across most datasets. Some categories of the CULane dataset have shown worse results, especially the night frames, curved roads, and crossroads. The obtained results suggest that the future fork should be focused on improving the algorithm performance in case of hardly visible lane markings (in situations like at night), in case of curved roads, and in the presence of complex markings (like crossroads).

Additionally, although the execution times of the proposed SMPPALD are on an acceptable level in terms of real-time processing, execution times of the algorithm could be additionally improved by implementing multithreaded solutions to deal with multiple lane markings in parallel. To ensure the usefulness of SMPPALD, as part of future work, it will also be implemented on multiple other segmentation NNs to verify the increase in performance in relation to the NN itself.

## Figures and Tables

**Table 1:** Resolutions and number of frames in the training, validation, and testing sets in the three datasets used. Testing set in Llamas is crossed out as it does not contain ground truth and was not used for testing.

Dataset	Frame Resolution	Number of Training Frames	Number of Validation Frames	Number of Testing Frames
CULane [7]	1640 × 590	88,880	9675	34,680
TuSimple [10]	1280 × 720	3626	358	2782
Llamas [11]	1276 × 717	58,269	20,844	20,929 (unused)

**Table 2:** Testing categories contained within the CULane testing set.

CULane Test Category	Number of Frames
Normal	9621
Crowd	8113
Night	7029
No Line	4067
Shadow	930
Arrow	890
Dazzle	486
Curve	422
Crossroad	3122

**Table 3:** PSO parameters specified in the post-processing algorithm, as well as their numerical types and descriptions.

PSO Parameter	Numerical Type	Description
contrast	Float	Contrast multiplier (alpha)
brightness	Float	Brightness (beta)
gamma	Float	Gamma to use for the LUT preprocessing
histCrop	Int	Amount of bottom to crop for histogram calculations
histThresh	Float	The threshold above which the marking detection finds the marking
clashArea	Float	The X area in which a repeat of the search is needed, if two markings end up as the same
windowWidth	Int	Width of a sliding window
windowHeight	Int	Height of a sliding window
outlierFirst	Float	The maximum X distance of the initial window from the marking detection-found starting point to be rejected as an outlier
outlierStart	Float	The outlier value from the first found window
outlierIncr	Float	The accumulated increment of the outlier when windows are skipped
minLineDots	Float	Minimum number of found dots in a window for it to be considered
thresholdMin	Float	Minimum threshold needed for the window to be considered
thresholdDip	Float	Subtracted from the found max threshold for the window
thresholdFirst	Float	The threshold needed when starting a marking from the bottom
xLineOverlap	Float	How close a marking overlaps to be removed
overlapCheck	Int	ID of the point to use when checking for overlaps
yLineExtraTop	Int	Extra extension for the top
yLineOverTop	Float	The height above which the upper extend will be calculated
yLineSpacing	Int	How many points to skip when generating a marking
edgeDistance	Int	Distance from edge
yMinDistance	Int	Minimum distance allowed between point heights on output

**Table 4:** Ranges of the PSO parameters in relation to the datasets.

PSO Parameter	Range for CULane	Range for TuSimple	Range for Llamas
contrast	[1, 2]	[1, 4]	[0.5, 3]
brightness	[−70, −30]	[−70, 0]	[−80, 0]
gamma	[5, 20]	[5, 20]	[2, 20]
histCrop	[80, 120]	[70, 110]	[110, 180]
histThresh	[5, 25]	[5, 25]	[10, 30]
clashArea	[5, 25]	[2, 15]	[2, 15]
windowWidth	[20, 60]	[20, 60]	[30, 70]
windowHeight	[5, 20]	[5, 20]	[5, 30]
outlierFirst	[5, 30]	[10, 40]	[20, 50]
outlierStart	[5, 30]	[5, 30]	[20, 50]
outlierIncr	[0.5, 4]	[0.2, 3]	[0.5, 8]
minLineDots	[5, 25]	[5, 25]	[5, 25]
thresholdMin	[40, 80]	[40, 80]	[40, 80]
thresholdDip	[20, 50]	[10, 40]	[5, 30]
thresholdFirst	[60, 100]	[60, 100]	[60, 120]
xLineOverlap	[10, 40]	[10, 40]	[10, 50]
overlapCheck	[0, 3]	[0, 3]	[0, 3]
yLineExtraTop	[1, 5]	[1, 5]	[5, 10]
yLineOverTop	[80, 120]	[80, 120]	[40, 100]
yLineSpacing	[1, 3]	[1, 3]	[1, 3]
edgeDistance	[0, 20]	[0, 20]	[0, 10]
yMinDistance	[10, 20]	[5, 20]	[2, 10]

**Table 5:** Comparison of results between the SCNN post-processing and the results of SMPPALD. All values are in F1 measure, except for the CULane (Crossroad), which is represented in the number of False Positive (FP) detections. Better results are marked in bold.

Dataset	SCNN Post-Processing F1 Measure (or FP)	SMPPALD F1 Measure (or FP)
CULane (Normal)	**0.906**	0.897
CULane (Crowd)	0.697	**0.703**
CULane (Night)	**0.661**	0.597
CULane (No Line)	0.434	**0.465**
CULane (Shadow)	0.669	**0.695**
CULane (Arrow)	0.841	**0.851**
CULane (Dazzle)	0.585	**0.603**
CULane (Curve)	**0.644**	0.611
CULane (Crossroad)	**1990 (FP)**	2263 (FP)
TuSimple	0.887	**0.915**
Llamas	0.913	**0.936**

**Table 6:** Comparison of SMPPALD frame processing execution times for different datasets. Provided are the shortest, longest, mean, and median execution times.

Dataset	Shortest Time	Longest Time	Mean Time	Median Time
CULane (Test set)	56.52 ms	467.21 ms	112.77 ms	112.38 ms
TuSimple (Test set)	65.25 ms	675.65 ms	188.95 ms	180.34 ms
Llamas (Validation set)	78.86 ms	693.61 ms	250.2 ms	245.17 ms

## Data Availability

No new data were created or analyzed in this study. Data sharing is not applicable to this article.

## Abbreviations

The following abbreviations are used in this manuscript:

ADAS	Advanced Driver Assistance System
LD	Lane Detection
NN	Neural Network
ROI	Region Of Interest
IOU	Intersection Over Union
PSO	Particle Swarm Optimization
